# Rational Design of Protein C Activators

**DOI:** 10.1038/srep44596

**Published:** 2017-03-15

**Authors:** Sergio Barranco-Medina, Mary Murphy, Leslie Pelc, Zhiwei Chen, Enrico Di Cera, Nicola Pozzi

**Affiliations:** 1 Edward A. Doisy Department of Biochemistry and Molecular Biology, Saint Louis University School of Medicine, St. Louis, MO 63104 USA

## Abstract

In addition to its procoagulant and proinflammatory functions mediated by cleavage of fibrinogen and PAR1, the trypsin-like protease thrombin activates the anticoagulant protein C in a reaction that requires the cofactor thrombomodulin and the endothelial protein C receptor. Once in the circulation, activated protein C functions as an anticoagulant, anti-inflammatory and regenerative factor. Hence, availability of a protein C activator would afford a therapeutic for patients suffering from thrombotic disorders and a diagnostic tool for monitoring the level of protein C in plasma. Here, we present a fusion protein where thrombin and the EGF456 domain of thrombomodulin are connected through a peptide linker. The fusion protein recapitulates the functional and structural properties of the thrombin-thrombomodulin complex, prolongs the clotting time by generating pharmacological quantities of activated protein C and effectively diagnoses protein C deficiency in human plasma. Notably, these functions do not require exogenous thrombomodulin, unlike other anticoagulant thrombin derivatives engineered to date. These features make the fusion protein an innovative step toward the development of protein C activators of clinical and diagnostic relevance.

The human body maintains a careful balance between bleeding and clotting. The master regulator of this equilibrium is thrombin, a trypsin-like protease essential for life^1^. Thrombin is generated upon initiation of the coagulation cascade from the inactive precursor prothrombin and plays a procoagulant role by cleaving fibrinogen to form a fibrin clot and a prothrombotic role by cleaving PAR1 to activate platelets. In addition, thrombin controls its own generation by initiating the so called protein C (PC) pathway with the assistance of the cofactor thrombomodulin (TM) and the endothelial protein C receptor (EPCR)^2^. Binding of TM prevents cleavage of fibrinogen and PAR1 while facilitating the productive encounter with PC. The thrombin-thrombomodulin complex accelerates conversion of PC ~2000-fold compared to thrombin alone^2^,^3^,^4^,^5^,^6^,^7^. *In vivo*, the reaction is further enhanced ~10-fold by EPCR, which binds and localizes PC to the endothelium near TM^2^,^8^. Activated protein C (aPC) cleaves and inactivates factors Va and VIIIa with the assistance of protein S and down regulates the amplification and progression of the coagulation cascade. In addition, aPC provides cytoprotective and regenerative activities by signaling through PAR1 and PAR3 in ways that differ completely from thrombin’s activation mechanism^9^. Deficiency of PC is linked to neonatal purpura fulminans^10^, which is fatal unless treated by replacement therapy. Mild deficiency^11^ or impaired activation of PC due to genetic disorders^12^ lead to an increased risk to develop venous thromboembolism. Even low levels of aPC due to limited availability of thrombomodulin have been documented in life-threatening conditions such as atherosclerotic lesions, stroke, sepsis, and disseminated intravascular coagulation^13^,^14^,^15^,^16^.

Because of PC’s essential physiological roles as an anticoagulant and anti-inflammatory factor, efforts have long been devoted to the development of efficient PC activators^17^. Protac, a toxin extracted from the snake venom *Agkistrodon contortix contortix*, was discovered in 1985^18^,^19^,^20^ and remains the gold standard for detection and quantification of PC in human plasma. Since then, other PC activators have been isolated from snake venoms but their activity is often promiscuous^21^. Furthermore, snake venoms find no use in clinical practice due to scarce availability, toxicity and allergenic properties. In the human body, thrombin’s activity represents the major source of aPC with other factors like meizothrombin^22^,^23^,^24^ and clotting factor Xa^25^ making additional but much smaller contributions. Infusion of thrombin is slightly anticoagulant but eventually prothrombotic^26^, which precludes any clinical application. Recombinant thrombin variants with selective specificity towards PC have been engineered^27^,^28^,^29^,^30^,^31^ and the double mutant W215A/E217A (WE) is currently moving to Phase I clinical trials for the treatment of thrombotic emergencies and stroke^30^,^32^,^33^. Proven advantages of PC activators such as WE over exogenous administration of aPC include reduced risk of bleeding^30^,^33^, unique antiplatelet activity via inhibition of the GPIb-von Willebrand factor (vWF) interaction^34^ and enhanced cytoprotective signaling at the level of the endothelium^35^. Though promising, these existing PC activators have a major weakness insofar as they fail to generate pharmacological quantities of aPC in clinical settings where TM is down-regulated or inactivated by proteases^13^,^14^,^15^,^16^. The absolute requirement for TM also precludes use of such variants as diagnostic tools alternative to Protac.

To overcome these limitations, we engineered proteins where the N-terminal of the EGF456 domain of TM is fused to the C-terminal of thrombin through a peptide linker of variable length. These innovative thrombin-thrombomodulin fusion proteins (FPs) feature efficient PC activation with only modest activity toward procoagulant and proinflammatory substrates and hold much promise as clinical and diagnostic tools.

## Results

### Rational Design of Thrombin-Thrombomodulin Fusion Proteins (FPs)

TM is a type I transmembrane protein that contains a large extracellular region, a single transmembrane sequence, and a short intracellular domain^13^,^14^. The extracellular region is composed of an N-terminal lectin-like domain, followed by six epidermal growth factor (EGF) modules, and a serine/threonine-rich domain. The minimal functional unit required for PC activation has been localized to the fourth through the sixth EGF domains (TM456)^13^,^36^. EGF5 and EGF6 bind to exosite-I of thrombin with high affinity^37^. EFG4 is dispensable for the interaction with thrombin but is absolutely required for cofactor activity^38^. TM456 can be expressed as a soluble protein and so is thrombin, which can be produced by autoactivation^39^ or limited proteolysis from the zymogen precursors prothrombin, prethrombin-1 or prethrombin-2. Thrombin-Thrombomodulin Fusion Proteins (FPs) were obtained by cloning the fragment TM456 of TM (V345-G465) after the C-terminal of thrombin using an appropriate DNA gene block. Proper connection between the two modules was ensured by an amino acid linker composed of Gly, Ser and Ala residues^40^. The small size of these amino acids allows for mobility of the connecting functional domains, reduces unfavorable interactions between the linker and protein moieties and minimizes immunogenicity of the linker^40^,^41^. A total of six FPs with a linker composed of 7, 17, 31, 41, 55 and 69 amino acids were produced in baby hamster kidney cells to identify the optimal length that would allow proper intramolecular complex formation. Initially, thrombin wild-type was fused to TM456 and the kinetics of cleavage of PC, fibrinogen and an extracellular fragment of PAR1 were used to establish if FPs recapitulate the properties of thrombin bound to TM.

### Functional characterization of FPs

**Figure 1:**
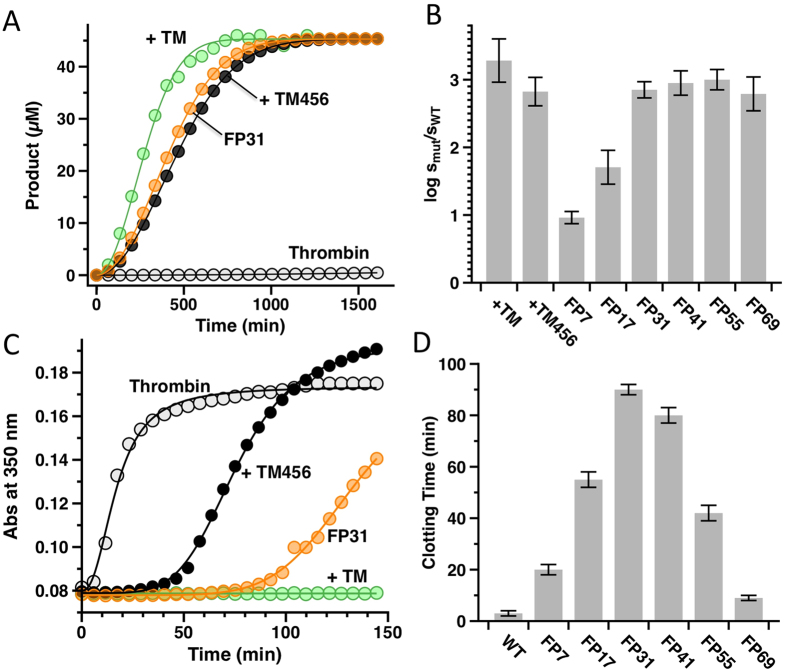
Protein C activation (**A,B**) and clotting of fibrinogen (**C,D**) by wild-type thrombin and FPs. (**A**) Shown are progress curves of D-DRR-pNA hydrolysis by aPC generated with thrombin alone (0.1 nM, light gray), thrombin in complex with 10 nM TM (green), 500 nM TM456 (black) and FP31 (orange). PC concentration was 50 nM. Experimental conditions are 5 mM Tris, 0.1% PEG, 145 mM NaCl, and 5 mM CaCl_2_, pH 7.4 at 37 °C. Progress curves were analyzed as described earlier^3^ to retrieve the values of specificity (*k*_*cat*_/K_M_) reported in Table 1. In the absence of TM, the activity of thrombin is negligible. Addition of TM, TM456 or fusing TM456 to thrombin rescues the activity of the enzyme. (**B**) Shown is the change in the specificity constant (s = *k*_*cat*_/K_M_) due to mutation, expressed as log s_mut_/s_wt_, under experimental conditions of 5 mM Tris, 0.1% PEG, 145 mM NaCl, and 5 mM CaCl_2_, pH 7.4 at 37 °C. Each FP was tested at a concentration of 0.1 nM. The values of specificity extracted from analysis of the progress curves are also reported in Table 1 and are the average of 3 independent determinations. (**C**) As fibrinogen is converted to fibrin the light is scattered through the fibers and the signal can be recorded at 350 nm. Clotting curves were obtained for thrombin alone (0.3 nM, light gray), in complex with 10 nM TM (green), 500 nM TM456 (black) and FP31 (orange). The presence of TM456 either added to the solution or fused to the enzyme prolonged but did not abolish the ability of thrombin to clot fibrinogen. (**D**) A similar effect was obtained with FPs but the magnitude of such effect was dependent on the length of the peptide linker. FP31 and FP41 were more effective than 500 nM exogenous TM456 whereas FP69 showed barely any effect. The other constructs displayed an intermediate behavior. The bell-shaped dependence on the length unequivocally proves that the interaction between EGF456 and thrombin is intramolecular (concentration-independent) and not intermolecular. From this analysis, maximum prolongation of the clotting time was achieved with a linker 31 residues long. Each FP was tested at a concentration of 0.3 nM and the values reported in Fig. 2Dare the average of three independent determinations.

TM binds to thrombin with a *K*_d_ = 1 nM^37^, but the interaction between TM456 and thrombin is 10–20 fold weaker due to lack of the chondroitin sulfate moiety attached to the serine/threonine rich domain^13^. PC is activated very slowly with thrombin alone (Fig. 1A), but addition of 10 nM TM increases the rate of cleavage ~2000-fold, from 0.12 to 230 ± 20 mM^−1^s^−1^. Saturating concentrations of TM456 (500 nM) enhance activation ~700-fold, from 0.12 to 80 ± 8 mM^−1^s^−1^, and are optimal in the presence of 0.5 mM Ca^2+^ as reported in previous studies^38^ (data not shown). All six FPs feature efficient PC activation under these same experimental conditions compared to thrombin alone (Fig. 1A,B and Table 1). A modest increase of 10–40 fold is observed with FP7 and FP17, but significant PC activation is obtained for FPs with a linker of 31 (85 ± 5 mM^−1^s^−1^), 41 (107 ± 8 mM^−1^s^−1^), 51 (120 ± 10 mM^−1^s^−1^) and 69 (74 ± 5 mM^−1^s^−1^) residues. Importantly, stimulation of activity toward PC by FPs is coupled with reduced activity toward fibrinogen and the extracellular fragment of PAR1. Under conditions where thrombin triggers clot formation in <5 min (Fig. 1C), no appreciable clotting is observed even after 150 min in the presence of 10 nM TM. Notably, saturating concentrations of TM456 (500 nM) significantly prolong but do not abrogate clotting. A similar effect is obtained with FPs (Fig. 1D, Table 1). The functional properties of FPs depend on the length of the linker. Activity toward PC follows a broad distribution centered on 50 residues (Fig. 1B). In contrast, activity toward fibrinogen and the extracellular fragment of PAR1 obeys a narrow distribution peaked at 31 residues (Fig. 1D). The difference is mechanistically significant and indicates that docking of TM456 on exosite-I of thrombin affects differently interaction with physiological substrates. The residual activity toward fibrinogen and the extracellular fragment of PAR1 should be considered in the context of the overlapping epitopes between these substrates and TM on exosite-I^42^,^43^,^44^. It has been proposed that mature circulating thrombin interacts with PAR1 and fibrinogen via exosite-I leading to rapid platelet activation, amplification of thrombin generation, and efficient conversion of fibrinogen to fibrin^38^,^42^,^45^,^46^,^47^,^48^. Binding of TM to exosite-I precludes docking of fibrinogen and PAR1 and accelerates the conversion of PC to aPC by facilitating the productive encounter between enzyme and substrate^3^,^6^,^7^,^44^,^49^. The observation that saturating concentrations of TM but not TM456 abrogate fibrinogen clotting and PAR1 cleavage shows that TM and EGF456 likely interact differently and/or with distinct domains of the thrombin surface.

### Optimization of FPs by site directed-mutagenesis: FP31_W215A_ and FP31_WE_

**Figure 2:**
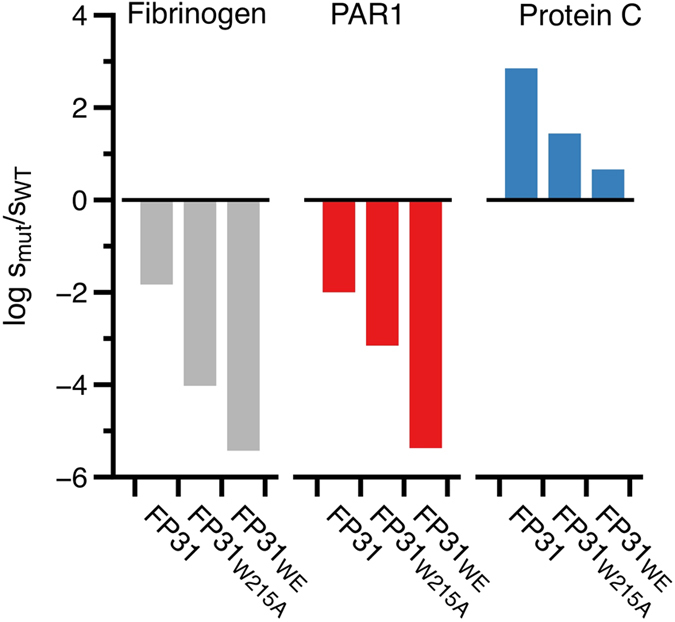
Optimization of the fusion proteins by site directed-mutagenesis. Shown is the change in the specificity constant (s = *k*_*cat*_/K_M_) for fibrinogen (gray), extracellular fragment of PAR1 (red) and PC activation (blue) due to mutation, expressed as log s_mut_/s_wt_. The values of specificity for thrombin wild-type are 17 μM^−1^s^−1^ for fibrinogen, 30 μM^−1^ s^−1^ for PAR1 and 0.12 mM^−1^s^−1^ for PC. Fusing TM456 to thrombin with a linker of 31 residues (FP31) reduces 60- and 100-fold the ability of thrombin to cleave fibrinogen and PAR1 respectively but stimulates PC activation. Substitution of W215 alone (FP31_W215A_) or W215/E217 (FP31_WE_) with alanine further decreases the activity toward fibrinogen and PAR1. Yet PC activation is retained. Data are reported in Table 1 and are the average of three independent determinations.

**Figure 3:**
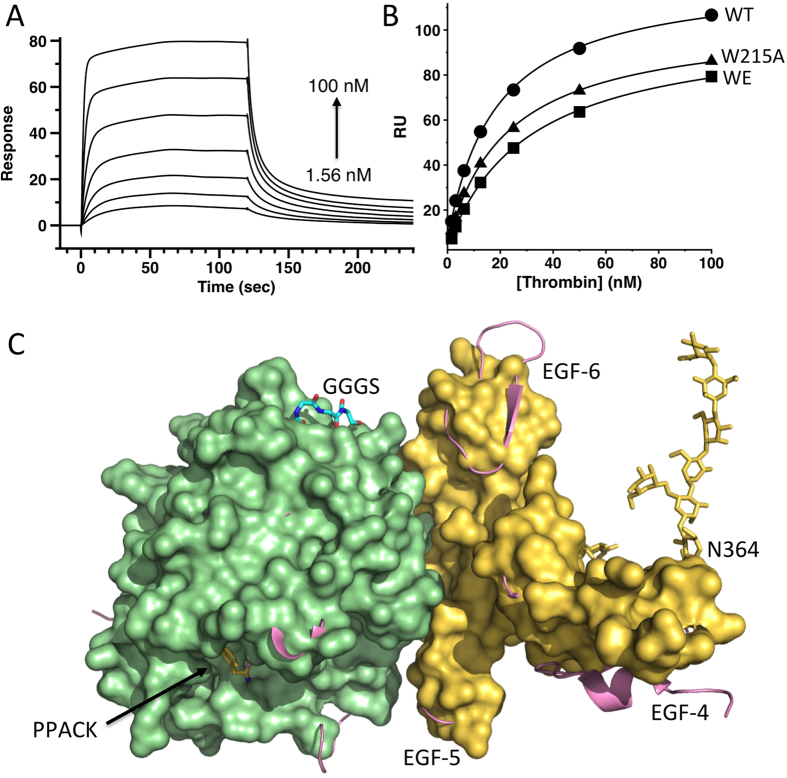
Biochemical and structural validation of the FP. Binding of thrombin wild-type (WT) and mutants W215A and WE to TM456 monitored by SPR. (**A**) Shown are the sensograms of the interaction between thrombin WE (1000–1.56 nM) and immobilized TM456. (**B**) Plot of response units (RU) as a function of the concentration of thrombin WT (solid circles), W215A (solid triangles) and WE (solid squares). Solid lines were drawn according to a simple binding equation with best-fit parameters listed in Table 2. (**C**) Crystal structure of FP31_WE_ showing a surface representation of WE (pale green) and EGF456 (gold) docked onto exosite I. Also shown are PPACK (yellow sticks) bound to the active site and a 7-unit sugar chain (gold sticks) covalently attached to N364 in EGF4. Only the initial GGGS sequence of the linker (cyan sticks) attached to the C-terminus of WE is visible in the density map, with the rest of the linker being completely disordered. The Cα-Cα distance between Ser in this linker and E346 in the N-terminus of EGF4 is 57 Å and long enough to accommodate the rest of the linker. The structure of thrombin bound to EGF456 of TM reported previously as 1DX5 is shown in cartoon representation (salmon) for comparison. The rmsd between the two structures is only 0.29 Å.

Residues W215 and E217 of thrombin are key to recognition of fibrinogen and PAR1 but nearly dispensable for PC activation^29^. The double thrombin mutant W215A/E217A (WE) features remarkable activity as an anticoagulant *in vitro* and *in vivo*^27^,^29^,^30^,^33^. To further reduce the activity of FP31 toward fibrinogen and the extracellular fragment of PAR1, we fused the thrombin mutants W215A and WE to EGF456 using a linker of 31 residues to generate FP31_W215A_ and FP31_WE_. The catalytic activity of FP31_W215A_ and FP31_WE_ toward fibrinogen dropped 2–4 orders of magnitude compared to FP31 (Fig. 2) and a significant reduction was also observed toward the extracellular fragment of PAR1. However, activity toward PC decreased 25–160 fold for FP31_W215A_ and FP31_WE_ compared to FP31. The drop argues for an inefficient allosteric communication between EGF456 and the active site of thrombin W215A or WE rather than incomplete mimicry of the TM456 interaction with exosite-I. In fact, the kinetic properties of FPs are recapitulated by the separate components in solution, yet differ from those obtained in the presence of TM (Table 2). Furthermore, direct SPR binding of TM456 to W215A and WE returns values of *K*_d_ comparable to those of wild-type thrombin (Fig. 3A,B). Correct assembly of the thrombin and EGF456 components of FP is confirmed by the X-ray crystal structure of FP31_WE_ bound to the active site inhibitor PPACK (Fig. 3C), that reproduces the bimolecular complex of thrombin and EGF456 reported previously^50^,^51^. Most of the contacts made by EGF45 with exosite I match the epitope validated by Ala scanning mutagenesis in previous studies^44^. Two glycosylation sites are clearly visible in the density map, with a single sugar attached to N391 and a long chain of 7 sugars attached to N364. Although dispensable for the co-factor activity^50^,^51^, the length of the glycosylation site at N364 is intriguing and suggests how EGF4 of thrombomodulin could steer PC toward the active site of thrombin. The linker connecting WE to EGF456 is only partially visible in the density map. Four residues attached to the C-terminus of WE remain 57 Å away from the N-terminus of EGF4, a distance that can easily accommodate the 27 residues of the linker missing in the density map.

### Effect of FPs on the activated partial thromboplastin time (aPTT)

**Figure 4:**
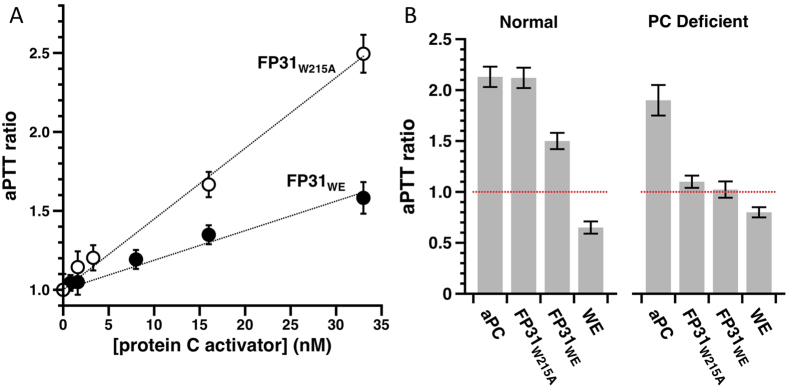
Effect of FPs on the aPTT. (**A**) In human plasma, FP31_W215A_ (empty circles) and FP31_WE_ (filled circles) increase aPTT linearly in a dose-dependent manner featuring an anticoagulant profile. (**B**) Unlike aPC (25 nM), the anticoagulant effect of FPs (25 nM) requires PC since no prolongation of the aPTT time was observed with PC depleted plasma (right panel). Due to the lack of soluble TM in normal and PC deficient plasma, thrombin WE (25 nM) features slightly reduced aPTT. aPTT values are shown as the ratios of the test to normal. Reference values are 39 ± 2 s for normal plasma and 42 ± 5 for PC depleted plasma. The dashed red line identify the baseline. Each measurement is the average of four individual determinations.

Despite their reduced activity toward PC, both FP31_W215A_ and FP31_WE_ promote an anticoagulant response in the absence of external TM. Addition of FPs to human plasma prolongs aPTT linearly in a dose-dependent manner (Fig. 4A). Incubation of 25 nM FPs with normal plasma (Fig. 4B, left panel) for 5 min returns an aPTT ratio of 2.1 for FP31_W215A_ and 1.5 for FP31_WE_, similar to the effect of added aPC but different from addition of WE. Importantly, FPs require circulating PC to prolong aPTT since PC depleted plasma is insensitive to the action of FPs but not of added aPC (Fig. 4B, right panel).

### Detection and quantification of the level of PC in human plasma

**Figure 5:**
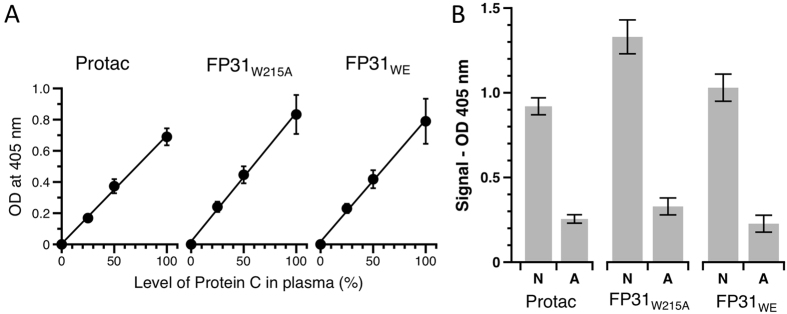
Detection and quantification of the level of protein C in human plasma. (**A**) Standard calibration curves were generated by replacing Protac with the FPs and following manufacturer’s instructions. Protac, FP31_W215A_ and FP31_WE_ efficiently convert PC to aPC, which is detected using S2366 as chromogenic substrate (Abs at 405 nm). (**B**) Randomized healthy (n = 6) and abnormal control plasma samples (n = 6) were tested side by side with Protac. As expected, depletion of PC from plasma results in a lower signal at 405 nm. Under these experimental conditions, FP31_W215A_ and FP31_WE_ performed better than Protac providing an improved N/A ratio, i.e. 4.5 for FP31_W215A_, 4.2 for FP31_WE_ and 3.6 for Protac.

FPs also afford diagnostic tools for the detection and quantification of the level of activity of PC in plasma, which currently relies on Protac. Currently, plasma is incubated for 5 min with Protac and the amount of aPC is detected using a chromogenic substrate. Quantification depends on a calibration curve using normal control plasma with known concentration of PC. We replaced Protac in this assay with WE, FP31_W5215A_ and FP31_WE_. WE failed to generate aPC in the absence of external TM, as expected (data not shown). In contrast both FPs showed a linear range up to 100% PC, with slopes of 0.0082 and 0.0078 for FP31_W215A_ and FP31_WE_, respectively (Fig. 5A). When mixed with randomized normal healthy (N: 95–130%) and abnormal (A: 30–46%) control plasma, FP31_W215A_ and FP31_WE_ gave remarkable N/A ratio (Fig. 5B) proving that both FPs, and especially FP31_W215A._ efficiently diagnose PC deficiency in human plasma.

## Discussion

The importance of the PC pathway in human pathophysiology is well established^2^ and so is the fundamental property of thrombin as a key enzyme in the blood capable of activating PC with the assistance of TM and EPCR. Interest in mimicking the action of TM on thrombin has led to the development of small molecules^52^ or of the more successful thrombin derivatives with selective activity toward PC^27^,^29^,^30^. One such mutant, thrombin WE, has shown remarkable activity *in vitro* and *in vivo*^30^, yet the absolute need for TM^29^,^53^ limits its diagnostic and therapeutic applications.

Physiological PC activators that obviate the need for endogenous TM could be used as diagnostic tools to detect and quantify the level of PC in plasma, a signature of PC deficiency and a potential biomarker for acquired thrombophilic disorders. As protein therapeutics, such molecules could find clinical use in thrombotic emergencies in which the level of aPC is down-regulated due to limited availability of TM^13^,^15^,^16^. Under these circumstances, direct oral anticoagulants and heparin have limited use because of the high risk of bleeding and lack of effective antidotes^54^. Infusion of recombinant aPC is no longer offered due to its uncertain risk-benefit profile (i.e. bleeding risk margin) after the PROWESS-SHOCK trial^55^,^56^. Non-anticoagulant versions of aPC are currently in clinical trials for ischemic stroke in combination with tPA, wound healing and retinal injury whereas sepsis and acute lung injury are not subject for current clinical studies^8^. Injection of recombinant soluble TM is efficacious in DIC^57^ but it is unclear whether its activity is manifested through PC activation, immunological functions or other unknown mechanisms.

This study reports an innovative step toward the development of PC activators that function in the absence of endogenous TM. FPs obtained by fusing thrombin to the functionally essential EGF456 fragment of TM via an amino acidic linker recapitulate most of the physiological properties of the thrombin-thrombomodulin complex. In these FPs, thrombin is always bound to EGF456 and no longer requires external TM for activity toward PC or abrogation of activity toward fibrinogen and PAR1. Functional and structural data support intramolecular docking of EGF456 on exosite-I of FPs and point to a strategy to optimize these constructs further. In human plasma, FPs show a significant anticoagulant profile. They prolong the aPTT by generating pharmacological quantities of aPC without external TM and without significant clotting of fibrinogen. FP31_WE_ is likely a better candidate for further development. Its procoagulant (fibrinogen) and proinflammatory (PAR1) activities are reduced 25- and 160-fold relative to FP31_W215A_, but PC activation is compromised only 6-fold. Any residual activity toward fibrinogen and PAR1 may eventually limit clinical applications requiring I.V. administration over extended periods of time. Preliminary data also show that FP31_WE_ is more resistant to ATIII inactivation compared to FP31_W215A_. Resistance to ATIII inhibition is expected to extend the half-life of FPs in the circulation, but this will require future PK studies in animal models.

FPs offer an intriguing alternative to Protac, which is used worldwide in clinical laboratories for the detection and quantification of PC in human plasma. Protac is a toxin extracted in low yield from snake venom and has limited availability. In contrast, FPs can be manufactured with a scalable and reproducible method in high yield, high purity and low cost. The mechanism of action of Protac remains elusive and contributes to false negative or positive results and misdiagnosis. For example, Protac based commercial assays fail to diagnose novel PC mutations like T315A and R229W^58^,^59^. Other potential advantages of FPs over current technologies are noteworthy. FPs may elevate the endogenous level of aPC independent of TM and should retain binding to the platelet receptor GPIb, thereby inhibiting GPIb-dependent interaction with vWF-collagen under shear^34^. Accumulation of FPs on platelet-rich thrombus may also promote local generation of aPC and increase local anticoagulant activity, an effect not produced by direct administration of recombinant aPC. This would allow administration of FPs at doses that sustain anticoagulation but minimize the risk of bleeding. Future studies will clarify how the pharmacological profile of FPs differs from WE, soluble TM and the WE-TM complex in relevant animal models of thrombosis and especially in mice defective for TM^53^.

## Methods

### Protein Expression and Purification

Plasmid-encoding human prethrombin-1 in pNUT containing an N-terminal HPC4 purification tag served as starting genetic material. cDNA encoding soluble EGF456 domains of human thrombomodulin (V363-G465) was fused before the ending codon of prethrombin-1 sequence using an appropriate gBlock (Integrated DNA Technologies, Coralville, IA) and megaprimer restriction-free (RF) cloning. At the junction between prethrombin-1 and TM456, linkers containing glycine, serine and alanine residues were introduced using the Quick-Change Lightning kit (Agilent, Santa Clara, CA) and appropriate primers (Integrated DNA Technologies, Coralville, IA). DNA constructs were verified by sequencing and transfected into baby hamster kidney (BHK) cells using Lipofectamine 3000 (Invitrogen, CA). The chemical composition of the linkers is: L7=GGGGGGG, L17=GGGSSSAGGGSSSGGGG, L31=GGGSSSAGGGSSSGGGGSSSAGGGSSSGGGG, L41=GGGSSSAGGGSSSGGGGSSSAGGGSSSGGGGASSSGSAGSS, L55=GGGSSSAGGGSSSGGGGSSSAGGGSSSGGGGASSSGSAGSSGGGGASSSGSAGSS, L69=GGGSSSAGGGSSSGGGGSSSAGGGSSSGGGGSSSAGGGSSSGGGGSSSAGGGSSSGGGGASSSGSAGSS. FPs were expressed in stable BHK mammalian cell lines at high yield (10–33 mg/l). Purification of the recombinant proteins was carried out by antibody-affinity chromatography followed by anion exchange chromatography. Zymogen to protease conversion was achieved by addition of the snake venom ecarin, or a mixture of ecarin and factor Xa for FP31_W215A_ and FP31_WE_. Upon completion, the activation mixture was loaded onto a Heparin-Sepharose column and eluted with a linear gradient of 0.1–0.6 M NaCl. The enzymes were >98% pure and >98% active, as judged by SDS-PAGE and active site titrations with PPACK. The fragment TM456 was produced by cloning the soluble EGF456 domains of human thrombomodulin (V363-G465) before the ending codon of human prothrombin in pDEST40. An additional thrombin cleavage site (PR↓LI) was introduced after the C-termini of prothrombin in the artificial linker connecting the two modules (ProT-Linker(PR↓LI)-TM456). After addition of ecarin, the proteolytic reaction was monitored by SDS-PAGE and TM456 was purified as described earlier^49^,^51^. Only for this construct (ProT-Linker(PR↓LI)-TM456), methionine at position 388 was replaced with a Leu to prevent possible oxidations during manipulations and purification^60^. To impede proteolysis, R456 and H457 were mutated to Gln and Ala. In addition, N364 and N391 were mutated to Ala to block N-glycosylation and reduce heterogeneity. The N-linked oligosaccharides have been previously shown to be unnecessary for efficient protein C activation by the thrombin-thrombomodulin complex^4^,^49^,^51^,^60^. Full-length rabbit thrombomodulin (TM), factor Xa (FXa), fibrinogen and protein C (PC) and activated protein C (aPC) were purchased from Hematologic Technologies, VT. All other chemicals were from Sigma-Aldrich, MO.

### Kinetic assays

Activation of PC by thrombin and FPs was carried out using a continuous assay in the presence of 0.1–2.5 nM enzyme, 50–200 nM PC, 45 μM of chromogenic substrate H-D-Asp-Arg-Arg-p-nitroanilide (D-DRR-pNA) specific for aPC, and when specified 50 nM TM or 0–1000 nM TM456^3^. Experiments were carried out in 20 mM Tris, pH 7.4, 145 mM NaCl, 5 mM CaCl_2_, 0.1% PEG 8000 at 37 °C. In the fibrinogen clotting assay, human fibrinogen was desalted on a G25 Zebaspin Column (ThermoFisher, USA) and eluted with 20 mM Tris, pH 7.4, 145 mM NaCl, 0.1% PEG 8000. The concentration of the desalted solutions was determined by measuring the absorbance at 280 nm (0.1% = 1.50 mg^−1^·cm^−1^). The turbidity (i.e. absorbance at 350 nm) of a fibrinogen solution (250 nM) was measured after addition of thrombin or FPs (0.3 nM) in a Cary-50 spectrophotometer. The release of fibrinopeptide A (FpA) and hydrolysis of a soluble extracellular fragment of human PAR1, ^33^ATNATLDPR↓SFLLRNPNDKYEPFWEDEEKN^62^ (where the arrow indicates the site of cleavage between Arg41 and Ser42) were followed by reverse-phase HPLC using the methods reported previously^39^.

### Surface Plasmon Resonance (SPR)

SPR measurements were carried out at 25 °C on a Biacore-S200 instrument (GE-Healthcare). TM456 was biotinylated and then coupled to a neutravidin coated CM5 chip. Briefly, 20 μL (16 mM) of a freshly prepared solution of Sulfo-NHS-LC-LC-Biotin (ThermoFisher, USA) were added to a solution of TM456 (50 μL, 1.3 mg/mL) in PBS. The mixture sat on ice for 3 hours before purification on a Superdex S200 to eliminate the unreacted reagent. The incorporation of the biotin was verified by western blot using an anti-biotin antibody (Sigma, MO). Biotin was incorporated specifically at the N-terminus since TM456 has no Lys residues. A biotinyl-TM456 solution (1 μg/ml) in 10 mM HEPES pH 7.4, 150 mM NaCl, 2.5 mM Ca^2+^, 0.05% Tween20 was then loaded for a total of 40 sec on the neutravidin-coated CM5 chip at a flow rate of 10 μl/min (177 RU). Titrations were performed by injecting increasing concentrations (0–100 nM) of thrombin wild-type, W215A and W215A/E217A over the chip at a flow rate of 30 μl/min, contact and dissociation time of 120 sec. Regeneration was achieved with a solution composed of 2.5 M NaCl, 5 mM EDTA. Each binding curve was subtracted for the corresponding baseline obtained on the reference flow cell. The dissociation constant (K_d_) for each titration was obtained as a fitting parameter by plotting the value of the response units at the steady state, after reaching equilibrium at each ligand concentration. Data analysis was performed using the BIAevaluation software and OriginPro 2015. Each titration was repeated 3 times.

### X-ray crystallography

Crystallization of FP31_WE_ bound to (D)-Phenylalanyl-prolyl-arginyl Chloromethyl Ketone (PPACK) was achieved at 20 °C by the vapor diffusion technique using an Art Robbins Instruments Phoenix liquid handling robot and mixing 0.3 μl of 30 mg/ml protein and 0.1 μl reservoir solution. Optimization of crystal growth was achieved by the hanging drop vapor diffusion method mixing 6 μl of 30 mg/ml protein with 2 μl of reservoir solution with 0.1 M HEPES, pH 7.0 and 20% PEG 8000. The crystal was grown at 20 °C in 1–2 weeks and was frozen in the solution of 0.1 M HEPES, pH 7.0, 20% PEG 8000 and 25% glycerol. Data were collected with a home source (Rigaku 1.2 kw MMX007 generator with VHF optics) Rigaku Raxis IV++ detector and were indexed, integrated and scaled with the HKL2000 software package^61^. The structure was solved by molecular replacement using MOLREP from the CCP4 suite^62^ and the structure of thrombin-thrombomodulin complex, protein Data Bank accession code 1DX5^50^ as search model. Refinement and electron density generation were performed with REFMAC from CCP4 suite. 5% of the reflections were randomly selected as a test set for cross-validation. The model building was performed in COOT^63^. Final refinement was finished by program PDB_REDO^64^. Ramachandran plots were calculated using PROCHECK^65^. Statistics for data collection and refinement are summarized in Table S1 in the Supplementary materials. Atomic coordinates and structure factors have been deposited in the Protein Data Bank (accession code 5TO3).

### Activated Partial Thromboplastin Time (aPTT)

aPTT was measured using the TriniCLOT aPTT kit on a ST4 semiautomated coagulometer (Diagnostica Stago, Gennevilliers, France). Briefly, 50 μl of human control normal plasma (Biophen normal control plasma ref A223201) or protein C deficient plasma (Biophen protein C deficient plasma ref ADP100A) were incubated with 50 μl of protein C activator (0–100 nM FP31_W215A_ or FP31_WE_), aPC (75 nM) or thrombin WE (75 nM) and 50 μl of reagent S for 5 min at 37 °C in the appropriate cuvettes. The reaction was started by adding 50 μl of 25 mM CaCl_2_.

### Detection of PC in human plasma

PC determination was performed according to manufacturer’s instructions (Biophen Protein C, Aniara Diagnostica, OH) with minor modifications. Briefly, 25 μl of human control normal plasma (Biophen normal control plasma ref A223201) diluted 1:1 with sterile saline solution (0.9% NaCl) were incubated with 50 μl of protein C activator (Protac, 2 μM FP31_W215A_ or 2 μM FP31_WE_) for 5 min at 37 °C in a 96-well plate. The amount of activated protein C was quantified by adding 125 μL of a solution of chromogenic substrate S-2366 (2.4 mM) (Diapharma Group, OH) and thrombin specific inhibitor argatroban (160 μM) (Sigma-Aldrich, MO) for 5 min at 37 °C. The reaction was quenched by addition of 150 μl citric acid and read at 405 nm. Mixing human control normal plasma with sterile saline solution to give 0, 25, 50 and 100% protein C generated standard calibration curves reported in Fig. 5A. Healthy control plasma was purchased from Innovative Research, MI and human abnormal control plasma depleted of protein C (ref A223301) was purchased from Biophen, Aniara Diagnostica, OH.

## Additional Information

**How to cite this article**: Barranco-Medina, S. *et al*. Rational Design of Protein C Activators. *Sci. Rep.* **7**, 44596; doi: 10.1038/srep44596 (2017).

**Publisher's note:** Springer Nature remains neutral with regard to jurisdictional claims in published maps and institutional affiliations.

## Supplementary Material

Supplementary Information

## Figures and Tables

**Table 1:** Kinetic rate constants (*k*_*cat*_/K_M_) for protein C activation (PC), fibrinogen (FpA) and PAR1 cleavage by thrombin wild-type (WT) and FPs.

	PC (mM^−1^s^−1^)	FpA (μM^−1^s^−1^)	PAR1 (μM^−1^sec^−1^)
**WT**	0.12 ± 0.02	17 ± 1	30 ± 3
**FP7**	1.1 ± 0.2	6.7 ± 0.8	5.8 ± 0.5
**FP17**	6.1 ± 0.8	1.55 ± 0.5	2.0 ± 0.1
**FP31**	85 ± 5	0.3 ± 0.1	0.3 ± 0.1
**FP41**	107 ± 8	0.6 ± 0.2	0.95 ± 0.21
**FP55**	120 ± 5	1.3 ± 0.3	2.3 ± 0.2
**FP69**	74 ± 7	11 ± 2	14 ± 2
**FP31**_**W215A**_	3.3 ± 0.1	0.0016 ± 0.0002^a^	0.021 ± 0.002^a^
**FP31**_**WE**_	0.55 ± 0.08	0.000063 ± 0.000009^a^	0.00013 ± 0.00001^a^

Experimental conditions are: 20 mM Tris, 0.1% PEG 8000, 145 mM NaCl and 5 mM CaCl_2_ for PC activation pH 7.4, at 37 °C, 0.1–2.5 nM enzyme and 50–200 nM PC.

^a^The concentration of FP31_W215A_ and FP31_WE_ for FpA and PAR1 release was 50 and 150 nM, respectively. The values are the average of four independent determinations.

**Table 2:** Kinetic rate constants (*k*_*cat*_/K_M_) for protein C activation and binding affinities of thrombin wild-type (WT), W215A/E217A and WE to TM456.

	+ TM (mM^−1^s^−1^)	+ TM456 (mM^−1^s^−1^)	K_d_ TM456 (nM)
**WT**	230 ± 20	80 ± 8	18 ± 3
**W215A**	40 ± 5	3.2 ± 0.2	23 ± 2
**W215A/E217A** (**WE**)	10 ± 2	0.38 ± 0.05	31 ± 3

Experimental conditions are: 20 mM Tris, 0.1% PEG 8000, 145 mM NaCl_2_ and 5 mM CaCl_2_ pH 7.4, at 37 °C for the PC activation assays. The concentrations of TM and TM456 were 50 and 500 nM, respectively. The values are the average of three independent determinations. Binding affinities (K_d_) were obtained by SPR. Each titration was repeated 3 times.
