# Supplementary material for: Rational Design of Protein C Activators
Source: Sci Rep. 2017 Mar 15;7:44596. doi: 10.1038/srep44596 (PMC5353707; doi:10.1038/srep44596)
Supplement: Supplementary Information [file srep44596-s1.pdf]

Supplementary Information

Rational Design of Protein C Activators

Sergio Barranco-Medina, Mary Murphy, Leslie Pelc, Zhiwei Chen, Enrico Di Cera* and Nicola Pozzi*

Table S1. Crystallographic data for Thrombin W215A/E217A -Thrombomodulin Fusion Protein linker 31 ($FP31_{WE}$) bound to PPACK

Buffer /salt	100 mM HEPES, pH 7.0
PEG	8000 (20%)
PDB ID	5TO3
**Data collection:**	
Wavelength (Å)	1.54
Space group	$C222_1$
Unit cell dimen sions (Å)	a=62.9
	b=162.2
	c=128.9
Molecules/asymmetric unit	1
Resolution range (Å)	40-2.35
Observations	161312
Unique observations	27169
Completeness (%)	96.7 (91.5)
R_{sym} (%)	10.6 (41.2)
I/s(I)	11.4 (2.2)
**Refinement:**	
Resolution (Å)	40-2.35
R_{cryst}, R_{free}	0.214, 0.255
Reflections (working/test)	25770/1388
Protein atoms	3242
Solvent molecules	93
PPACK/K^+Na^+	1/1/1
Rmsd bond lengths[a] (Å)	0.014
Rmsd angles[a] (°)	1.8
Rmsd DB ($Å^2$) (mm/ms/ss)[b]	2.35/2.60/3.53
<B> protein ($Å^2$)	47.1
<B> solvent molecules ($Å^2$)	40.0
<B> PPACK/K^+Na^+ ($Å^2$)	35.7/25.9/52.9
**Ramachandran plot:**	
Most favored(%)	99.7
Generously allowed (%)	0.3
Disallowed (%)	0

[a]Root-mean-squared deviation (Rmsd) from ideal bond lengths and angles and Rmsd in B-factors of bonded atoms. [b]mm, main chain-main chain; ms, main chain-side chain; ss, side chain-side chain.
